# Malignant priapism secondary from metastatic renal carcinoma: Case report: “Malignant Priapism from Kidney Neoplasm”

**DOI:** 10.1016/j.eucr.2023.102496

**Published:** 2023-07-03

**Authors:** I Wayan Yudiana, Ronald Sugianto, Ni Putu Ekawati, Eric Sibastian Hutauruk

**Affiliations:** a Department of Urology, Faculty of Medicine, Udayana University, Prof. Dr. I.G.N.G Ngoerah General Hospital, Bali, Indonesia; b Medical Doctor Study Program, Faculty of Medicine, Udayana University, Bali, Indonesia; c Department of Anatomical Pathology, Faculty of Medicine, Udayana University, Prof. Dr. I.G.N.G Ngoerah General Hospital, Bali, Indonesia; d Departement of Urology, Siloam Private Hospital, East Nusa Tenggara, Indonesia

**Keywords:** Penile metastases, Kidney neoplasm, Priapism, Distal shunt, Penectomy, Urethrostomy

## Abstract

Malignant priapism (MP) is defined as a condition of persistent erection of the penile without sexual stimulation due to malignant cell invasion to the cavernous sinus and the efferent veins. We present a case of a man, 63 years old, with previous history of high-grade renal carcinoma pT3N0M0 had been through radical nephrectomy, diagnosed with MP secondary from metastatic renal carcinoma. The management of this case was aspiration of corpora cavernosa and distal shunting with the Al-Ghorab procedure, then continued to total penectomy and perineostomy.

## Highlights

• Malignant priapism is a persistent erection due to malignant cell invasion.
• The origin of penile metastases is commonly urogenital cancers.
• Despite that fact, renal carcinomas as the primary tumor were rare.
• Prognosis for patients with malignant priapism was poor.

## 1 Introduction

Malignant priapism (MP), first described by Peacock in 1938, is defined as a condition of persistent erection of the penile without sexual stimulation due to malignant cell invasion to the cavernous sinus and the efferent veins.^1^ MP has been suggested as one of the clinical manifestations of penile metastases. The origin of penile metastases is commonly urogenital cancers (69%), followed by gastrointestinal cancer (18%).^2^ Although the pathogenesis caused by a malignancy is rare, it can explain the priapism onset with a history of malignancy.

## 2 Case presentation

**Fig. 1:**
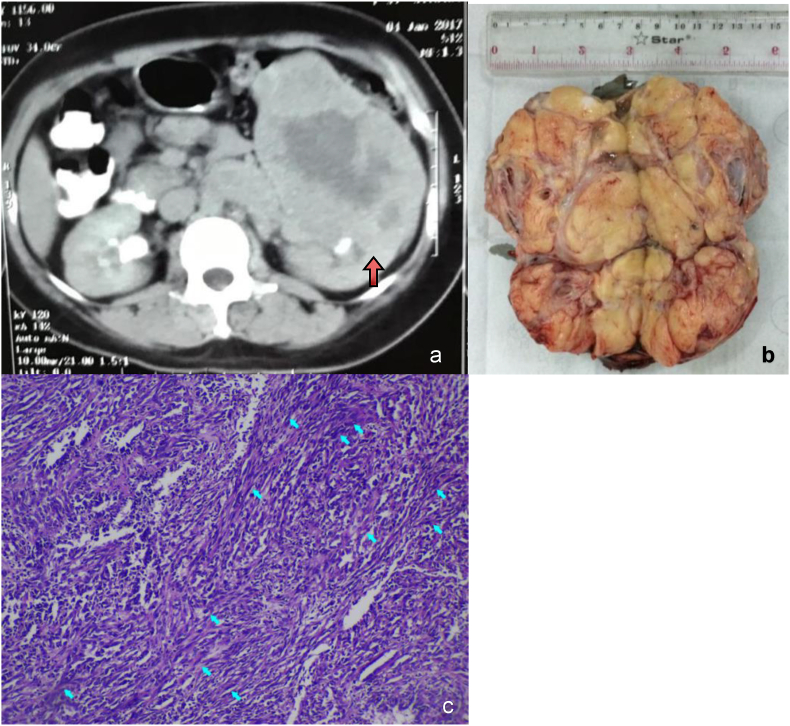
Diagnostic examination of renal carcinoma. a) Axial CT-Scan abdomen without contrast showed a left-sided renal tumor (red arrow). b) The left renal tumor was about 23 x 16 × 12 cm. c) Microscopic findings of spindle tumor cells (blue arrow). (For interpretation of the references to colour in this figure legend, the reader is referred to the Web version of this article.)

A male patient, 63 years old, came to the emergency department and complained of flank pain and a weight loss of about 8 kg in 2 months. The physical and laboratory examination found flank mass and pain. The following laboratory examination demonstrated anemia, leukocytosis, and decreasing renal function with hemoglobin 9.8 g/dL, microcytic and hypochromic erythrocytes, white blood cell counts 12.13 x 103/μL, estimated glomerular filtration rate 42.59 and creatinine 1.68 mg/dL. In suspicions of malignancy, CT-Scan was performed and provided a hyperdense mass in the left kidney with a central necrosis appearance, without thrombosis in the inferior vena cava and distant metastasis, as presented in Fig. 1a.

The patient underwent a radical nephrectomy then the mass went through pathological anatomy examinations, shown in Fig. 1b and c. The result demonstrated a high-grade carcinoma, a polymorphic type of renal cell carcinoma (RCC) in the left renal. As a result, the patient was diagnosed with high-grade renal carcinoma pT3N0M0.

Two weeks later, he complained of pain and prolonged erection without sexual stimulation for a week. The physical examination showed erection in the shaft penis with flaccidity in the gland penis. The clinical diagnosis for the patient was non-ischemic priapism. To detumescence the penile, corpora cavernosa was aspirated 10 ml, dark red blood, but the detumescence did not appear. Then, the treatment was through surgical management for distal shunting by the Al-Ghorab technique, but there was no significant reduction of the priapism.

**Fig. 2:**
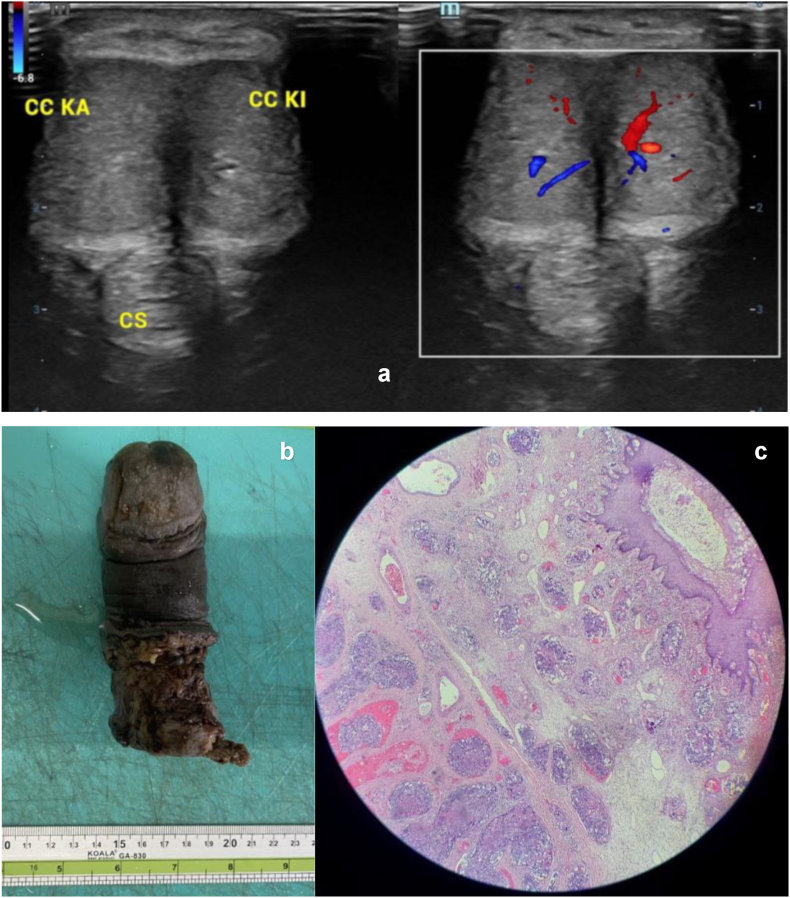
Diagnostic examination of MP. a) Penile doppler ultrasonography demonstrated non-ischemic or high-flow priapism. b) The resection of the penis was about 11 x 5 × 3.5 cm and covered by skin tissue along 6 cm. c) Microscopic findings of the infiltrative neoplastic cell into corpora cavernosa.

Based on the condition, further radiologic examinations were performed. The penile doppler ultrasonography showed non-ischemic priapism with suspicion of MP, multiple hypoechoic lesions with solid mass and calcification in the middle to the distal part of corpora cavernosa bilaterally, subcutis edema in the distal region of penile, and fluid collection in peri tunica albuginea bilateral, as presented in Fig. 2a. Inversely, the chest x-ray showed no abnormal result without any metastatic lesion. Therefore, the patient underwent a total penectomy and perineostomy. The pathologic anatomy examination was held after total penectomy to determine the cause of priapism, which showed a high-grade carcinoma infiltration into the penile gland, corpus spongiosum, and corpora cavernosa continued to the proximal part of the resection sample, shown in Fig. 2b and c. After the total penectomy, the patient is still regularly treated by adjuvant radiotherapy and tyrosine kinase inhibitor (pazopanib) for cancer cells in the margin of resection.

The patient followed up every month to assess the progression, the effectiveness of treatment, and the quality of life due to CaST. Finally, the patient has been followed-up for three months with no abnormalities in urination and no significant complaints. The case presentation followed the SCARE Guideline checklist.^3^

## 3 Discussion

The Color Doppler ultrasound has proven its efficiency in diagnosing and differentiating between the two types of priapism. In ischemic priapism, the cavernosal arteries and corpora cavernosa had a low or even absent blood flow, whereas in non-ischemic, the blood flow is normal or significantly increased.^4^ Further imaging investigations, such as CT-Scan, MRI, and internal pudendal/penile arteriography, were not performed in the initial phase of the evaluation. The investigation protocol for suspicion of malignancy should also include the assessment of tumor markers, bone marrow studies, and imaging investigations like thoracic, abdominal, and pelvic computer tomography evaluation.^5^

Our cases were uncommon, which were a non-ischemic type of MP. Although an infiltrative process of MP was commonly from urogenital cancer, renal cancer as the primary cancer sites were less common than the bladder and prostate as the primary cancer sites. The actual cause of MP remains unknown yet. The proposed hypotheses of non-ischemic MP were the development of an arterio-cavernosal fistula caused by an erosion of the cavernosal arteries and branches by infiltration of metastatic cancer.^5^ However, this hypothesis has not appeared in several case reports and our case.

According to EAU guidelines on Priapism 2022, the effectiveness of the conventional therapeutic recommendations was unlikely to be low. When previous treatments were not able to detumescence the penile or were inadequate to control pain, total penectomy can be performed.^4^ Therefore, some reports performed total penectomy to prevent the progression of carcinoma infiltration.

The prognostic of the penile metastases was different due to the origin of the cancer and the presentation of priapism. The analysis showed that the metastases from urological origin generally have a better prognosis than those with a non-urological and that patients presenting with MP had a worse prognosis than those without MP. Overall, the CaST for penile metastasis ranged from 5 to 30 months. The median of CaST was 14.5 months.^2^

We found that studies related to renal metastasis, especially among testicles and penile are still scarce. We recommend further research or investigation into the testicle symptoms associated with renal carcinoma.

## 4 Conclusion

The metastatic process of MP originated from renal cancer, as the primary cancer sites were uncommon. The prognostic for renal cancer with MP was getting worsen within months. Modalities of therapy for MP varied from medication treatment, non-surgical treatment, surgical treatment, and radiotherapy.

## Consent

The informed consent was written by the patient in the Indonesian language for further publication of this case report anonymously. A copy of the written consent is available for review by the Editor-in-Chief of this journal on request.

## Funding

None.

## Declaration of competing interest

The authors report no conflicts of interest.
